# Relationship between coronary flow reserve evaluated by phase-contrast cine cardiovascular magnetic resonance and serum eicosapentaenoic acid

**DOI:** 10.1186/1532-429X-15-106

**Published:** 2013-12-20

**Authors:** Shingo Kato, Kazuki Fukui, Junko Kawaguchi, Nao Ishii, Masashi Koga, Yuka Kusakawa, Ikuyoshi Kusama, Tatsuya Nakachi, Takeshi Nakagawa, Yasuo Terauchi, Kazuaki Uchino, Kazuo Kimura, Satoshi Umemura

**Affiliations:** 1 Department of Cardiology, Kanagawa Cardiovascular and Respiratory Center, 6-16-1 Tomiokahigashi, Kanazawa-ku, Yokohama, Kanagawa 236-0051, Japan; 2 Department of Endocrinology and Metabolism, Yokohama City University Hospital, Yokohama, Japan; 3 Department of Cardiology, Yokohama City University Hospital, Yokohama, Japan; 4 Division of Cardiology, Yokohama City University Medical Center, Yokohama, Japan

## Abstract

**Background:**

Long-term intake of long-chain n-3 polyunsaturated fatty acids (n-3 PUFAs), especially eicosapentaenoic acid (EPA) is associated with a low risk for cardiovascular disease. Phase-contrast cine cardiovascular magnetic resonance (PC cine CMR) can assess coronary flow reserve (CFR). The present study investigates the relationship between CFR evaluated by PC cine CMR and the serum EPA.

**Methods:**

We studied 127 patients (male, 116 (91%); mean age, 72.2 ± 7.4 years) with known or suspected coronary artery disease (CAD). X-ray coronary angiography revealed no significant coronary arterial stenoses (defined as luminal diameter reduction ≥50% on quantitative coronary angiogram (QCA) analysis) in all study participants. Breath-hold PC cine CMR images of the coronary sinus (CS) were acquired to assess blood flow of the CS both at rest and during adenosine triphosphate (ATP) infusion. We calculated CFR as CS blood flow during ATP infusion divided by that at rest. Patients were allocated to groups according to whether they had high (n = 64, EPA ≥ 75.8 μg/mL) or low (n = 63, EPA < 75.8 μg/mL) median serum EPA.

**Results:**

CFR was significantly lower in the low, than in the high EPA group (2.54 ± 1.00 vs. 2.91 ± 0.98, p = 0.038). Serum EPA positively correlated with CFR (R = 0.35, p < 0.001). We defined preserved CFR as > 2.5, which is the previously reported lower limit of normal flow reserve without obstructive CAD. Multivariate analysis revealed that EPA is an independent predictor of CFR > 2.5 (odds ratio, 1.01; 95% confidence interval, 1.00 – 1.02, p = 0.008).

**Conclusions:**

The serum EPA is significantly correlated with CFR in CAD patients without significant coronary artery stenosis.

## Background

Long-term intake of long-chain n-3 polyunsaturated fatty acids (n-3 PUFAs), especially eicosapentaenoic acid (EPA), is associated with a low risk for cardiovascular disease [1-7]. Several reports describe that n-3 PUFAs confer several benefits, such as antiarrhythmic effects [8,9] and the ability to reduce platelet aggregation [10,11] and stabilize coronary arterial plaque [12]. The GISSI-Prevenzione trial [13] revealed that dietary n-3 PUFA intake significantly prevented cardiovascular mortality in patients with a history of myocardial infarction. The Japan EPA Lipid Intervention Study (JELIS) [14] showed that concurrent therapy with purified EPA and statins reduces the incidence of coronary events.

Phase-contrast cine cardiovascular magnetic resonance (PC cine CMR) is a promising approach to quantifying global myocardial blood flow in the left ventricular (LV) myocardium without exposure to radiation [15-19]. The accuracy of this technique has been validated in phantoms [20], in animals using flow probes [18] and in humans using positron emission tomography (PET) [16]. Coronary flow reserve (CFR) calculated from CMR flow values in the coronary sinus at rest and during dipyridamole stress is significantly impaired in patients with hypertrophic cardiomyopathy [15], heart failure [21] and dilated cardiomyopathy [19].

Whether or not serum EPA levels correlated with the CFR of patients with known or suspected coronary artery disease (CAD) remains unclear. Therefore, the present study aimed to determine the relationship between serum EPA and CFR in patients with CAD using PC cine CMR. As the presence of significant coronary artery stenosis affects the CFR, we enrolled the CAD patients without ≥50% diameter stenosis on X-ray coronary angiography (CAG).

## Methods

### Patients

**Figure 1:**
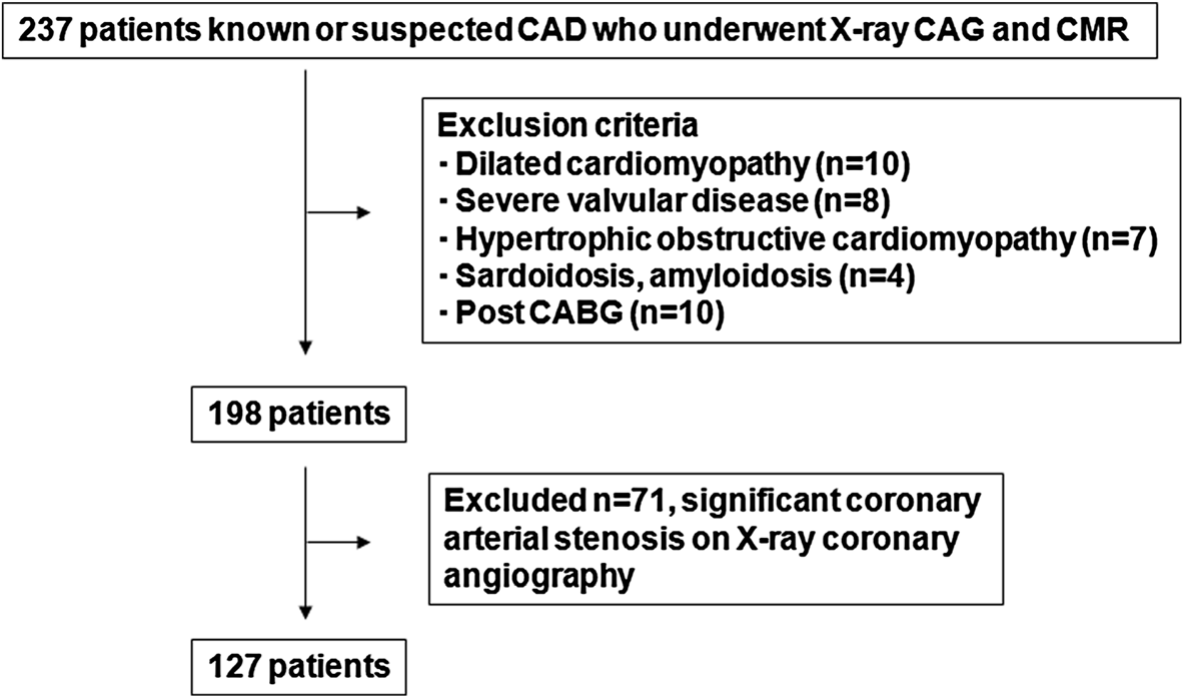
**Flow chart of enrollment of study population.** CAD, coronary artery disease; CMR, cardiovascular magnetic resonance; CABG, coronary artery bypass graft surgery.

This study included 237 patients with known or suspected CAD who were assessed by X-ray coronary angiogram and cardiovascular magnetic resonance (CMR) including cine CMR, PC cine CMR, late gadolinium enhancement (LGE) CMR. Figure 1 illustrates flow chart of patient enrollment in this study. We excluded the patients with dilated cardiomyopathy (n = 10), severe valvular disease (n = 8), hypertrophic cardiomyopathy (n = 7), sarcoidosis (n = 3) and amyloidosis (n = 1). We also excluded patients with history of coronary artery bypass graft surgery (CABG) (n = 10) and patients who demonstrated significant coronary arterial stenoses on X-ray CAG (n = 71). Finally, 127 patients (male, 116 (91%); mean age, 72.2 ± 7.4 years) were enrolled in the present study. Table 1 summarizes the characteristics of the included patients. The medical histories of 42 (33%), 64 (50%) and 72 (57%) of the patients included myocardial infarction, angina pectoris. We allocated the patients to groups with high (n = 64; EPA ≥ 75.8 μg/mL) and low (n = 63; EPA < 75.8 μg/mL) median serum EPA. Other characteristics including coronary risk factors, cardiovascular history and medication did not significantly differ between the groups (Table 1). None of them were taking purified EPA. All patients provided written, informed consent to participate in this study, which was approved by the local institutional review board.

**Table 1:** Patient characteristics

	**All patients**	**High EPA group**	**Low EPA group**	*P value
	**(n = 127)**	**(n = 64)**	**(n = 63)**	
Age	71.6 ± 7.8	71.8 ± 7.6	70.1 ± 9.1	0.18
Male	116 (91%)	60 (94%)	56 (89%)	0.12
Height, cm	163.4 ± 7.1	162.9 ± 7.7	164.4 ± 6.5	0.29
Body weight, kg	65.4 ± 11.1	65.9 ± 11.7	64.3 ± 9.7	0.51
Body mass index, kg/m^2^	24.3 ± 3.0	24.6 ± 3.2	23.7 ± 2.5	0.69
Systolic BP, mmHg	137.9 ± 20.8	135.7 ± 19.2	140.5 ± 22.3	0.24
Diastolic BP, mmHg	70.7 ± 12.6	69.6 ± 13.3	71.8 ± 11.9	0.35
Heart rate, beats per minute	67.9 ± 12.4	66.3 ± 10.7	69.6 ± 13.8	0.18
Cardiovascular history				
Myocardial infarction	42 (33%)	19 (30%)	23 (37%)	0.64
Angina pectoris	64 (50%)	35 (55%)	29 (46%)	0.22
**Coronary risk factors**				
Hypertension	93 (73%)	47 (73%)	46 (73%)	0.88
Dyslipidemia	92 (72%)	46 (72%)	46 (73%)	0.84
Diabetes Mellitus	39 (31%)	20 (31%)	19 (30%)	0.89
Current smoking	16 (13%)	9 (14%)	7 (11%)	0.48
Family history of CAD	6 (5%)	3 (5%)	3 (5%)	0.92
Obesity	41 (32%)	25 (39%)	16 (25%)	0.13
**Blood test results**				
EPA, μg/mL	85.3 ± 53.9	121.5 ± 53.3	48.4 ± 16.9	<0.001
DHA, μg/mL	139.1 ± 54.4	163.2 ± 51.9	109.0 ± 35.2	<0.001
AA, μg/mL	178.6 ± 52.9	171.0 ± 47.3	179.6 ± 52.5	0.34
Total cholesterol, mmol/L	179.0 ± 38.6	183.1 ± 36.7	172.8 ± 37.9	0.12
LDL cholesterol, mmol/L	101.9 ± 26.7	106.6 ± 31.7	100.2 ± 27.5	0.23
HDL cholesterol, mmol/L	57.3 ± 15.9	66.1 ± 64.0	53.0 ± 14.3	0.12
Triglyceride, mmol/L	179.0 ± 38.6	171.1 ± 125.1	157.6 ± 105.6	0.51
Hemoglobin, g/dL	14.3 ± 1.6	14.3 ± 1.4	14.1 ± 1.6	0.58
HbA1c, %	6.0 ± 0.9	6.0 ± 1.0	5.8 ± 0.8	0.21
eGFR, mL/min/1.73 m^2^	65.7 ± 14.0	64.5 ± 13.2	65.6 ± 13.6	0.65
CRP, mg/dL	0.30 ± 1.0	0.15 ± 0.21	0.42 ± 1.37	0.25
BNP, pg/dl	53.3 ± 65.0	46.6 ± 43.9	45.9 ± 63.2	0.94
**Medication use**				
Statin	82 (65%)	41 (64%)	41 (65%)	0.86
Antiplatelet agent	108 (85%)	52 (81%)	56 (89%)	0.91
Calcium channel blocker	45 (35%)	22 (34%)	23 (37%)	0.99
β blocker	48 (38%)	21 (33%)	27 (43%)	0.26
ACE/ARB	73 (57%)	33 (52%)	40 (63%)	0.17

Values are presented as mean ± standard deviation (SD).

* P value represents significance of difference between low EPA group and high EPA group.

EPA, eicosapentaenoic acid; AA, arachidonic acid, DHA, docosahexaenoic acid; BP, blood pressure; CAD, coronary artery disease; LDL; low density lipoprotein; HDL, high density lipoprotein, eGFR, estimated glomerular filtration rate; CRP, C-reactive protein; BNP, brain natriuretic peptide; ACE, angiotensin converting enzyme inhibitor; ARB angiotensin receptor blocker.

### CMR acquisition

CMR was performed on a 1.5-T MR system equipped with 32 channel cardiac coils (Achieva, Philips Healthcare, Best, The Netherlands). All patients were assessed by cine CMR, PC cine CMR and LGE CMR. Imaging was performed after overnight fasting. Serum fatty acid measurement was performed on the same day as CMR.

### Cine CMR acquisition

Vector-electrocardiographic (VCG) monitoring leads were positioned on supine patients and then imaging started. Scout images were acquired in three orthogonal planes for cardiac orientation. Vertical and horizontal long-axis cine CMR of the LV was acquired using a steady-state free precession (SSFP) sequence. The LV volume and mass were calculated from short-axis cine images of the LV acquired from the apex to the base (repetition time, 4.1 ms; echo time, 1.7 ms; flip angle, 55°; field of view, 350 × 350 mm; acquisition matrix, 128 × 128; slice thickness, 10 mm; and number of phases per cardiac cycle, 20).

### Phase-contrast cine CMR acquisition

Cine CMR in the axial plane was obtained through the atrioventricular groove to locate the coronary sinus (Figure 1). The imaging plane for blood flow measurement by PC cine CMR was positioned perpendicular to the coronary sinus 2 cm from the ostium of the coronary sinus. Phase-contrast cine CMR of the coronary sinus was acquired during suspended shallow breath-holding using a VCG triggered gradient echo sequence (repetition time, 7.3 ms; echo time, 4.4 ms; flip angle, 10°; field of view, 240 × 194 mm; acquisition matrix, 128 × 128; and number of phases per cardiac cycle, 20). Pharmacological stress was achieved by injecting ATP (160 μg · kg^–1^ · min^–1^) into the left antecubital vein for 4 min. PC cine CMR images of the coronary sinus were acquired during ATP stress and at rest. The duration between stress and resting image acquisition was at least 10 min. All patients were asked to refrain from caffeinated beverages for at least 24 hours prior to CMR.

### Late gadolinium enhanced image acquisition

After acquiring phase-contrast cine CMR, the patients were injected with 0.15 mmol/kg of gadopentetate dimeglumine (Magnevist, Bayer Healthcare, Leverkusen, Germany). Late gadolinium-enhanced CMR was obtained in the same planes as cine images 15 min later using an inversion recovery–prepared gradient-echo sequence. The imaging parameters for late gadolinium-enhanced imaging comprised: repetition time, 4.3 ms; echo time, 1.3 ms; flip angle, 15°; field of view, 380 × 380 mm; acquisition matrix, 256 × 180; and slice thickness, 10 mm. The null point of the normal myocardium was determined using a Look-Locker sequence.

### X-ray coronary angiography

An observer who was blinded to the results of CMR interpreted the conventional X-ray CAG using quantitative software (QangioXA, Medis, Inc., Raleigh, North Carolina). Intracoronary administration of isosorbide dinitrate (2 to 3 mg) was performed in all patients before contrast injection. We performed quantitative coronary angiography (QCA) analysis for evaluating the degree of coronary arterial stenosis on X-ray CAG. In our trial, significant coronary arterial stenosis was defined as reduction in luminal diameter ≥50% by QCA analysis.

### Measurement of serum fatty acids

Blood was sampled from all patients after an overnight fast for at least 12 h. Serum levels of EPA, AA, and docosahexaenoic acid (DHA) were measured using a gas chromatograph (GC-2010; Shimadzu, Kyoto, Japan) and a capillary column (BPX90; Wako, Osaka, Japan) at a central laboratory (BML, Tokyo, Japan).

### Data analysis

Two observers used a workstation (Extend MR WorkSpace, Philips Healthcare) to analyze the cine MR, PC cine MR, and LGE images. All CMR images were rendered anonymous and reviewed in random order. To measure the LV cardiac mass, epi- and endocardial borders of the LV on the short axis cines were manually traced with exclusion of the papillary muscles at each anatomic level that encompassed the LV. The LV mass was calculated by the consensus of two observers as the sum of the myocardial volume areas multiplied by the specific gravity (1.05 g/mL) of the myocardial tissue [22].

The contours of the coronary sinus were manually traced on each frame of all PC cine images to quantify blood flow in the coronary sinus and velocity in the adjacent tissue was measured for phase-offset correction. Blood flow in the coronary sinus was calculated by integrating the product of the cross-sectional area and the mean velocity in the coronary sinus and corrected using mean velocity in the adjacent tissue for all cardiac phases in the cardiac cycle. In line with other studies, we also corrected coronary sinus blood flow using rate pressure products (RPP) [16,19,23-26].

$$\begin{array}{ll} \;\mathrm{RPP}\;\left(\mathrm{mmHg}‧\mathrm{bpm}\right) & =\mathrm{Systolic}\;\mathrm{blood}\;\mathrm{pressure}\;\left(\mathrm{mmHg}\right)\\ & \;\times \mathrm{Heart}\;\mathrm{rate}\;\left(\mathrm{beats}/\min\right)\\ \;\mathrm{Corrected}\;\mathrm{CS}\;\mathrm{flow} & \left(\mathrm{mL}/\min\right)=\mathrm{CS}\;\mathrm{flow}\;\left(\mathrm{mL}/\min\right)\\ & \;/\mathrm{RPP}\;\left(\mathrm{mmHg}‧\mathrm{bpm}\right)\times 7500 \end{array}$$

The ΔCS flow and CFR were calculated as:

$$\begin{array}{ll} \Delta \mathrm{CS}\;\mathrm{flow}\left(\mathrm{mL}/\min\right)= & \mathrm{Corrected}\;\mathrm{CS}\;\mathrm{flow}\;\mathrm{during}\;\mathrm{ATP}\\ & \mathrm{infusion}\;\left(\mathrm{mL}/\min\right)-\mathrm{Corrected}\\ & \mathrm{CS}\;\mathrm{flow}\;\mathrm{at}\;\mathrm{rest}\;\left(\mathrm{mL}/\min\right) \end{array}$$

$$\begin{array}{ll} \mathrm{Coronary}\;\mathrm{flow}\;\mathrm{reserve}\;\left(\mathrm{CFR}\right)= & \mathrm{Corrected}\;\mathrm{CS}\;\mathrm{flow}\;\mathrm{during}\\ & \mathrm{ATP}\;\mathrm{infusion}\;\left(\mathrm{mL}/\min\right)\\ & /\mathrm{Corrected}\;\mathrm{CS}\;\mathrm{flow}\;\mathrm{at}\\ & \mathrm{rest}\;\left(\mathrm{mL}/\min\right) \end{array}$$

The coronary vascular resistance (CVR) was calculated as follows.

$$\begin{array}{l} \begin{array}{ll} \;\mathrm{CVR}\;\left(\mathrm{mmHg}‧\min/\mathrm{mL}\right)= & \mathrm{mean}\;\mathrm{arterial}\;\mathrm{pressure}\\ & \left(\mathrm{MAP}\right)\;\left(\mathrm{mmHg}\right)/\mathrm{corrected}\\ & \mathrm{CS}\;\mathrm{flow}\;\left(\mathrm{mL}/\min\right) \end{array}\\ \begin{array}{ll} \;\mathrm{MAP}\;\left(\mathrm{mmHg}\right)= & \left[\mathrm{systolic}\;\mathrm{BP}\;\left(\mathrm{mmHg}\right)+2x\;\mathrm{diastolic}\;\right)\\ & \left(\mathrm{BP}\;\left(\mathrm{mmHg}\right)\right]/3 \end{array} \end{array}$$

Late gadolinium-enhancement was visually assessed and interpreted by the consensus of two observers who were blinded to the patients’ information. The mass with LGE was measured on short-axis slices using manual planimetry. The total mass of LGE was calculated by summing the late gadolinium-enhanced mass of all sections, and the ratio (%) of LGE is expressed as:

$$\begin{array}{ll} \;\mathrm{LGE}\;\left(\%\right)= & \mathrm{Total}\;\mathrm{LGE}\;\mathrm{volume}/\mathrm{Total}\;\mathrm{LV}\;\mathrm{wall}\;\mathrm{volume}\\ & \times 100\;\left(\%\right) \end{array}$$

None of the patients had atypical enhancement defined as mid-myocardial or sub-epicardial enhancement with multifocal distribution.

### Statistical analysis

Data were statistically analyzed using SPSS software, version 17.0 (SPSS, Inc., Chicago, IL, USA). Continuous values are presented as means ± standard deviation (SD). Skewed values are presented as medians with the interquartile range (IQR). Normality was determined by the Shapiro-Wilk test. Coronary flow reserve determined by CMR was compared between the high and low EPA groups. Differences between the groups were evaluated using an unpaired *t*-test for normally distributed variables, and the Mann–Whitney U test for skewed variables. The relationship between serum fatty acids and CFR determined by CMR was determined using Pearson’s correlation coefficient. Preserved CFR was defined as CFR > 2.5 by PC cine CMR, which is the lower limit of normal flow reserve without obstructive CAD [27]. Independent predictors of impaired CFR were evaluated using univariate analysis and multivariate stepwise analyses. Inter- and intra-observer reproducibility was evaluated using the intraclass correlation coefficient (ICC). P values < 0.05 were considered statistically significant. In addition, we allocated study population into 4 groups based on presence or absence of DM and serum EPA. We evaluated significance of difference between 4 groups by using one-way analysis of variance (ANOVA).

## Results

### Cardiac CMR findings

**Figure 2:**
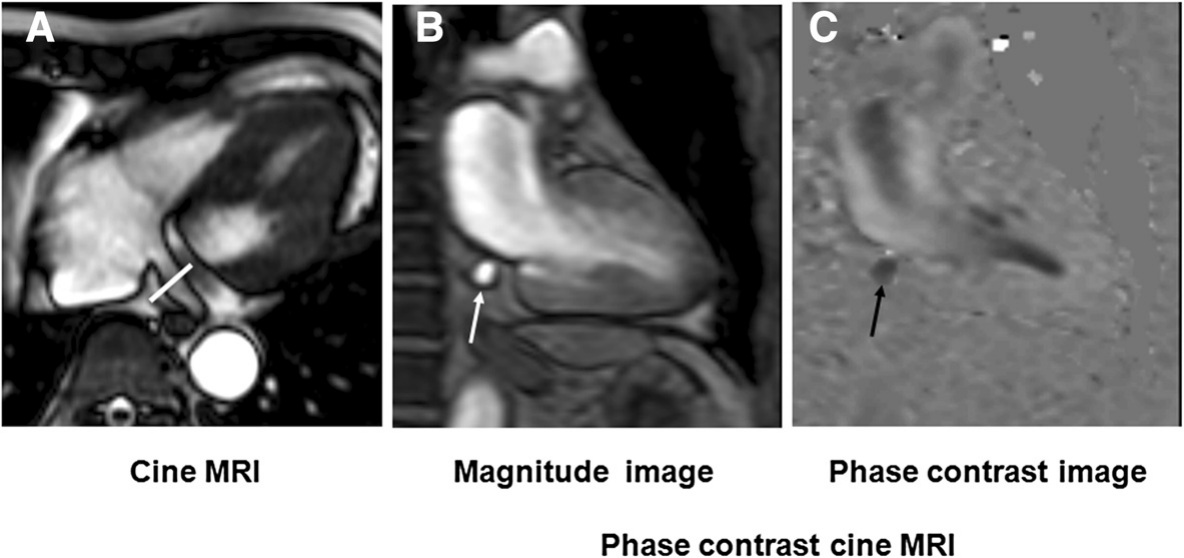
**Phase-contrast cine cardiovascular magnetic resonance of the coronary sinus. (A)** Axial image of the coronary sinus acquired by steady state free precession (white solid line). **(B)** Magnitude image of coronary sinus (white arrow). **(C)** Phase difference image of coronary sinus. Blood flow in the coronary sinus appears as a low signal intensity area in **(C)**, (black arrow). CMR, cardiovascular magnetic resonance.

**Figure 3:**
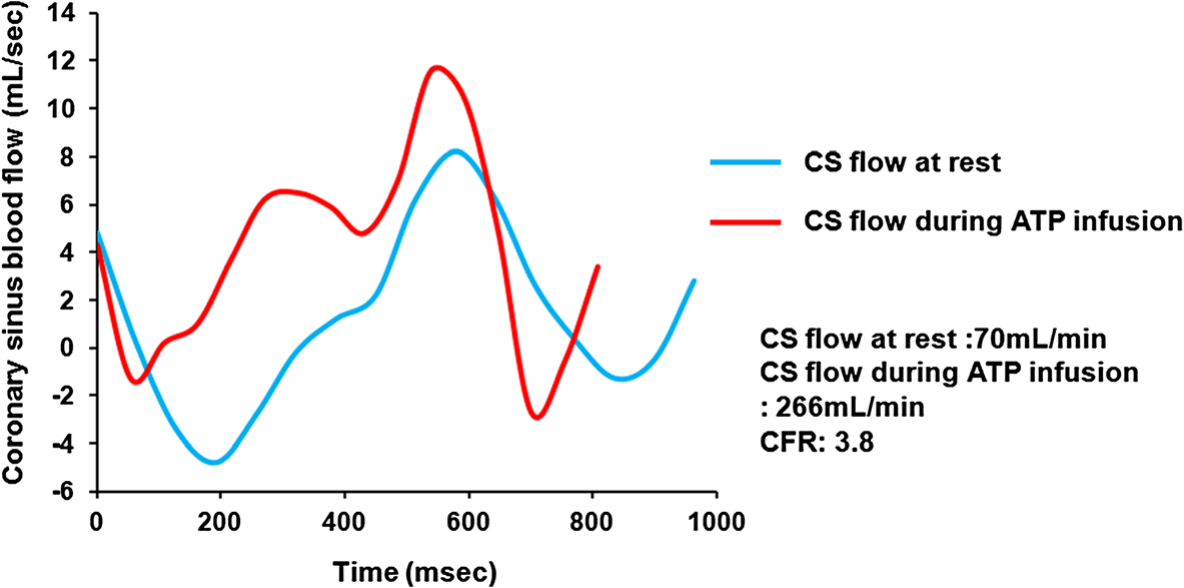
**Curves of blood flow in coronary sinus.** Blood flow was measured using phase-contrast cine CMR. Blue and red lines, blood flow at rest and during ATP infusion, respectively. Coronary sinus blood flow increased about 4-fold in this patient during ATP infusion (rest vs. ATP infusion: = 70 vs. 266 mL/min; CFR = 3.8). ATP, adenosine triphosphate; CFR, coronary flow reserve; CS, coronary sinus.

We acquired PC cine CMR from all patients in the resting state and during ATP stress (Figure 2). Figure 3 shows representative blood flow curves of the coronary sinus at rest and during infusion. Table 2 summarizes the CMR findings. Cine parameters such as LV end-diastolic volume index (LVEDVI), LV end-systolic volume index (LVESVI), LV ejection fraction (LVEF), LV mass index did not significantly differ between the low and high EPA groups. In addition, LGE parameters, such as presence of LGE and percentage of LGE (%) also did not significantly differ between the groups.

**Table 2:** CMR findings

	**All patients**	**High EPA group**	**Low EPA group**	* P value
	**(n = 127)**	**(n = 64)**	**(n = 63)**	
Cine CMR				
LVEDVI, mL/m^2^	73.9 ± 24.9	71.7 ± 21.8	76.1 ± 27.7	0.33
LVESVI, mL/m^2^	33.2 ± 19.9	31.0 ± 19.1	35.4 ± 20.7	0.21
LVSVI, mL/m^2^	40.8 ± 10.5	40.8 ± 8.6	40.8 ± 12.3	0.98
LVEF, %	57.1 ± 12.1	58.9 ± 11.7	55.3 ± 16.9	0.094
LV mass index, g/m^2^	56.3 ± 16.9	57.8 ± 17.0	54.8 ± 16.9	0.32
Late Gadolinium enhanced CMR				
Presence of LGE	66 (52%)	32 (50%)	34 (54%)	0.65
LGE (%)	10.6 ± 14.2	9.0 ± 13.1	12.2 ± 15.2	0.21

Values are presented as mean ± standard deviation (SD).

* P value represents significance of difference between low EPA group and high EPA group.

EPA, eicosapentaenoic acid; CMR, cardiovascular magnetic resonance; LV, left ventricle; EDVI, end-diastolic volume index; ESVI, end-systolic volume index; SVI, stroke volume index; EF, ejection fraction; LGE, late gadolinium enhancement.

### Comparison of coronary flow reserve in low and high EPA groups

Table 3 compares coronary sinus blood flow at rest and during ATP infusion, and CFR in the low and high EPA/AA groups. The coronary sinus blood flow measurements were reproducible, with ICCs of 0.93 and 0.95 for inter- and intra-observer reproducibility, respectively. No significant difference between low and high EPA groups was found in HR, SBP, DBP and RPP at rest and during ATP infusion. SBP and DBP were significantly decreased by ATP infusion both in low and high EPA groups. Corrected coronary sinus blood flow was significantly augmented by ATP infusion in the low and high EPA groups (from 79.1 ± 32.4 to 191.5 ± 87.2 mL/min, p < 0.001 and from 82.3 ± 35.0 to 229.5 ± 95.0 mL/min, p < 0.001, respectively), whereas ΔCS flow was significantly lower in the group with low EPA (112.4 ± 73.7 vs. 147.2 ± 77.7 mL/min, p = 0.036). Furthermore, CFR was significantly reduced in the low, compared with the high EPA group (2.54 ± 1.00 vs. 2.91 ± 0.98, p = 0.038). We found significant difference in CVR during ATP infusion between high and low EPA groups (0.53 ± 0.25 vs. 0.44 ± 0.26, p = 0.049). In addition, we divided study population into 4 groups based on presence or absence of DM and serum EPA (group 1, n = 44: DM(-) & EPA ≥75.8 μg/mL, group 2, n = 44: DM(-) & EPA <75.8 μg/mL, group 3, n = 20: DM (+) & EPA ≥75.8 μg/mL, group 4, n = 19: DM (+) & EPA <75.8 μg/mL. CFR was highest in group 1 and lowest in group 4 (mean CFR: 3.04 ± 1.00 in group1, 2.69 ± 1.11 in group2, 2.67 ± 1.04 in group 3, 2.39 ± 0.83). However, difference between 4 group did not show significant difference (p = 0.16 by one-way ANOVA).

**Table 3:** Coronary sinus blood flow at rest and during ATP infusion in groups with low and high EPA

	**All patients, (N = 127)**	**High EPA group, (n = 64)**	**Low EPA group, (n = 63)**	* P-value
HR at rest, bpm	68 ± 12	66 ± 11	70 ± 14	0.14
HR during ATP infusion, bpm	71 ± 12	69 ± 11	73 ± 12	0.086
SBP at rest, mmHg	138 ± 21	136 ± 19	140 ± 22	0.22
SBP during ATP infusion, mmHg	117 ± 20**	117 ± 19**	117 ± 22**	1.00
DBP at rest, mmHg	71 ± 13	69 ± 13	72 ± 12	0.33
DBP during ATP infusion, mmHg	62 ± 12**	62 ± 13**	72 ± 12**	0.87
RPP at rest	9432 ± 2505	9047 ± 2206	9825 ± 2739	0.11
RPP during ATP infusion	8303 ± 2105**	8116 ± 2111**	8508 ± 2100**	0.34
cCS flow at rest (mL/min)	77.4 ± 32.7	82.3 ± 35.0	79.1 ± 32.4	0.59
cCS flow during ATP infusion (mL/min)	203.3 ± 89.6**	229.5 ± 95.0**	191.5 ± 87.2**	0.021
Δ cCS flow (mL/min)	125.9 ± 75.8	147.2 ± 77.7	112.4 ± 73.7	0.011
Coronary flow reserve	2.75 ± 1.03	2.91 ± 0.98	2.54 ± 1.00	0.038
CVR at rest, mmHg‧min/mL	1.37 ± 0.54	1.33 ± 0.60	1.41 ± 0.47	0.73
CVR during ATP infusion mmHg‧min/mL	0.49 ± 0.26**	0.44 ± 0.26**	0.53 ± 0.25**	0.049

Data are expressed as mean ± SD. * P-value represents significance of the difference between low EPA group and high EPA group. ** P < 0.05 vs. at rest.

HR = heart rate, ATP = adenosine triphosphate. SBP = systolic blood pressure, DBP = diastolic blood pressure, RPP, rate pressure product; ATP, adenosine triphosphate; EPA, eicosapentaenoic acid; CS, coronary sinus, CVR = coronary vascular resistance.

cCS flow = corrected coronary sinus flow, Ι cCS flow = cCS flow during ATP infusion – cCS flow at rest. Coronary flow reserve = cCS flow during ATP infusion / cCS flow at rest × 100. MAP = (SBP + DBPx2)/3, CVR = MAP/cCS flow.

### Relationship of serum fatty acid and coronary flow reserve

**Figure 4:**
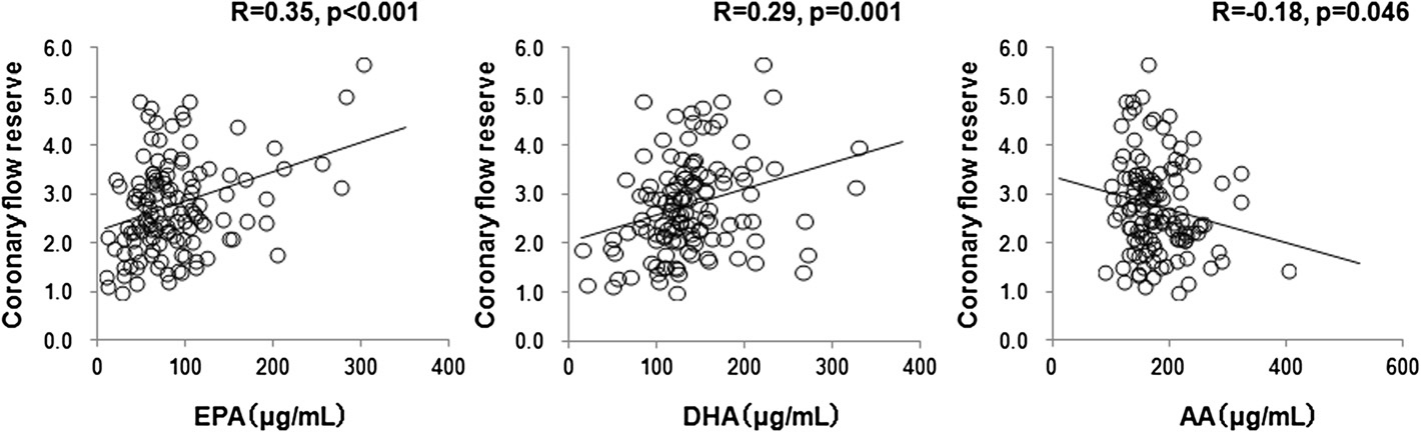
**Relationship between serum fatty acid and coronary flow reserve evaluated by phase-contrast cine cardiovascular magnetic resonance.** Serum levels of EPA and DHA significantly and positively correlate with CFR (R = 0.35, p < 0.001; R = 0.29, p = 0.001). Serum AA levels significantly and negatively correlate with CFR determined by PC cine CMR (R = -0.18, p = 0.046). CFR coronary flow reserve; DHA, docosahexaenoic acid; EPA, eicosapentaenoic acid.

Figure 4 shows relationships between serum fatty acids (EPA, DHA, AA) and CFR evaluated by CMR. Serum levels of EPA and DHA correlated significantly and positively (R = 0.35 and p < 0.001, R = 0.29 and p = 0.001, and R = 0.35 and p < 0.001, respectively), whereas those of AA correlated significantly and negatively (R = -0.18, p = 0.046) with CFR.

### Univariate and multivariate analyses of predictors of reduced CFR

Table 4 shows the results of the univariate and multivariate analyses of predictors of CFR > 2.5 determined by PC cine CMR. Univariate analysis selected history of diabetes mellitus, serum EPA, LVMI on cine CMR as significant predictors of CFR > 2.5. Variables with p values < 0.05 in the univariate analysis were included in the multiple stepwise regression models. Table 4 shows that the EPA was a significant determinant CFR > 2.5 (odds ratio (OR), 1.01; 95% confidence interval (CI), 1.00 – 1.02; p = 0.008). Other independent variables comprised history of diabetes mellitus (OR, 0.39; 95%CI, 0.17 – 0.89; p = 0.026), LVMI (OR, 0.97; 95%CI, 0.95 – 0.99; p = 0.024).

**Table 4:** Predictors of coronary flow reserve >2.5 determined by univariate and multivariate analyses

	**Univariate analysis**			**Multivariate analysis**		
	**Odds ratio**	**95% CI**	**P value**	**Odds ratio**	**95% CI**	**P value**
**Patients’ characteristics**						
Age, per year	1.01	0.97 – 1.05	0.65	Not selected		
Male, men	1.58	0.43 – 5.68	0.49	Not selected		
Systolic blood pressure, per mmHg	0.99	0.97 – 1.01	0.99	Not selected		
Diastolic blood pressure, per mmHg	0.98	0.95 – 1.02	0.31	Not selected		
Heart rate. Per bpm	0.98	0.95 – 1.01	0.19	Not selected		
History of myocardial infarction , yes	1.27	0.57 – 2.86	0.55	Not selected		
History of angina pectoris, yes	1.56	0.71 – 3.56	0.26	Not selected		
**Coronary risk factors**						
Hypertension, yes	0.69	0.26 – 0.69	0.45	Not selected		
Dyslipidemia, yes	0.67	0.25 – 1.77	0.42	Not selected		
Diabetes Mellitus, yes	0.35	0.16 – 0.77	0.009	0.39	0.17 - 0.89	0.026
Current smoking, yes	1.49	0.50 – 4.45	0.48	Not selected		
Family history of CAD, yes	1.21	0.23 – 6.40	0.82	Not selected		
Obesity, BMI ≥ 25 kg/m^2^, yes	1.41	0.66 – 3.00	0.37	Not selected		
**Blood testing**						
EPA, per μg/mL	1.01	1.00 – 1.02	0.017	1.01	1.00 – 1.02	0.008
Total cholesterol, per mmol/L	1.00	0.99 – 1.01	0.62	Not selected		
LDL cholesterol, per mmol/L	1.00	0.99 – 1.01	0.93	Not selected		
HDL cholesterol, per mmol/L	1.02	0.99 – 1.04	0.21	Not selected		
Triglyceride, per mmol/L	1.00	0.99 – 1.00	0.97	Not selected		
HbA1c, per%	0.92	0.63 – 1.34	0.68	Not selected		
eGFR, per mL/min/1.73 m^2^	0.99	0.97 – 1.03	0.94	Not selected		
CRP, per mg/dL	0.57	0.18 – 1.80	0.34	Not selected		
BNP, per pg/dl	1.00	0.99 – 1.01	0.66	Not selected		
**Cine CMR**						
LVEDVI, per mL/m^2^	1.00	0.99 – 1.01	0.75	Not selected		
LVESVI, per mL/m^2^	0.99	0.98 – 1.01	0.32	Not selected		
LVEF, per%	1.03	1.00 – 1.06	0.066	Not selected		
LVMI, per g/m^2^	0.97	0.95 – 1.00	0.019	0.97	0.95-0.99	0.024
**Late Gadolinium enhanced CMR**						
Presence of LGE, yes	0.65	0.32 – 1.32	0.24	Not selected		
LGE (%), per%	0.97	0.95 – 1.02	0.35	Not selected		

The variables with p value < 0.05 in the univariate analysis were included in the multivariate analysis.

CI, confidence interval; CFR, coronary flow reserve; LGE, late gadolinium enhancement; BMI, body mass index; EPA, eicosapentaenoic acid; LDL; low density lipoprotein; HDL, high density lipoprotein, eGFR, estimated glomerular filtration rate; CRP, C-reactive protein; BNP, brain natriuretic peptide; CMR, cardiovascular magnetic resonance; LV, left ventricle; EDVI, end-diastolic volume index; ESVI, end-systolic volume index; SVI, stroke volume index; EF, ejection fraction; LVMI, left ventricular mass index.

## Discussion

This is the first investigation of the relation between CFR evaluated as changes in coronary sinus flow induced by ATP infusion, and serum EPA. In patients without significant coronary arterial stenosis on X-ray coronary angiography, serum EPA and DHA levels significantly and positively correlated with CFR. Multivariate linear regression analysis showed that the EPA was an independent predictor of impairment of CFR evaluated by CMR. These results indicated that the serum EPA plays an important role in the regulation of CFR in patients with CAD.

### The independent predictors and potential mechanism of impairment of CFR

The results of multivariate regression analysis in this study revealed that presence of DM, LVMI and serum EPA were the independent predictors of preserved CFR (CFR > 2.5). The CFR is an index of coronary microvascular function in patients without epicardial coronary arterial stenosis, therefore, we assessed the relationship between microvascular function and serum EPA concentration. It’s well known that the women more frequently develop microvascular disease (MVD) in comparison to men, however, men and women who have coronary MVD often have conventional risk factors, such as hypertension and DM [28]. The presence of DM was an independent predictor of CFR in this study, and the result is in line with previous study. However, presence of hypertension and presence of dyslipidemia were not independent predictors of CFR. The main reason is that the ACEI/ARB treatment for hypertension or statin treatment for dyslipidemia was already started. These drugs potentially have benefirial effects to MVD [28]. The serum EPA might play an important role for the regulation of CFR, and EPA drug treatment potentially improve the microvascular function in patients with MVD.

LV hypertrophy is another important mechanism of CFR impairment. presence of significant coronary arterial stenosis that results in dilatation already at rest. The patients with LV hypertrophy have a higher level of auto-regulated blood flow at rest to meet the demand of an increased myocardial mass [29]. Therefore, CFR is reduced because resting flow is already increased closer to the maximal level. The LVMI was an independent predictor of CFR in the current study, which is in line with previous study.

### Clinical significance of serum fatty acid on CAD prevention

Epidemiological studies have shown that the incidence of acute myocardial infarction is substantially lower among non-emigrated inhabitants of Greenland (5.3%), than in individuals from the United States (40.4%) and Denmark (34.7%), with age- and sex adjusted death rates for ischemic heart disease [30]. This is mainly due to the composition of Eskimo food in north western Greenland, which is dominated by n-3 PUFA, such as EPA and DHA (ratios of n-3 PUFA in total Eskimo and Danish dietary intake of fatty acids: 13.7% and 2.8%, respectively) [30]. The randomized controlled GISSI-Prevenzione trial showed that dietary n-3 PUFA intake can reduce the rate of cardiovascular death among patients after a recent (≤ 3 months) myocardial infarction [13]. The large, randomized JELIS control trial found that that administering pure EPA together with statin therapy reduced the risk of developing coronary events to 19% [14]. However, precisely how n-3 PUFA prevents CAD remains unknown. Some reports have shown favorable effects of n-3 PUFA on the cardiovascular system. Leaf et al. showed that n-3 PUFA helped to prevent arrhythmic deaths, including sudden cardiac death [31]. Ghio et al. showed that n-3 PUFA significantly increases LV systolic function in patients with symptomatic heart failure of any etiology [31]. In addition, Moertl et al. found that n-3 PUFA administered for 3 months exerts a dose-dependent increase in the LVEF of patients with chronic heart failure. Moreover, high-dose n-3 PUFA intervention significantly improves endothelial function and decreases interleukin 6 levels [32]. We found that the serum EPA is an independent predictor of impaired CFR evaluated by PC cine CMR. This finding indicates that improved CFR might be an important mechanism through which n-3 PUFA prevents cardiovascular events.

### Clinical implications

In this study, we enrolled the CAD patients without ≥50% diameter stenosis on X-ray CAG, and assessed the microvascular function by calculating the CFR by PC-cine CMR. It is important to evaluate the disease severity in patients with non-obstructive CAD, because the prognosis of MVD is not as benign as commonly thought [28]. Randomized placebo-controlled studies have demonstrated that angiotensin-converting enzyme inhibitors, statins etc. relieve symptoms, vascular dysfunction, or both [28]. In the current study, most of our patients were administered with aspirin and statins, and had relatively low LDL cholesterol levels. Regardless, the EPA significantly correlated with CFR, and it was an independent predictor of reduced CFR. These results suggest that EPA therapy will help to improve CFR and prevent cardiovascular events through different mechanisms from statins or antiplatelet drugs. A further prospective interventional study is required to clarify this point.

### Study limitation

First, this was a single center study with a relatively small patient cohort. Although CFR assessment by PC cine CMR is non-invasive and useful, CMR is contraindicated for patients implanted with pacemakers or cardiovascular defibrillators and those with claustrophobia. Such patients were excluded from the present study. Second, we excluded the CAD patients with significant coronary arterial stenosis (diameter reduction ≥50% by QCA analysis). Therefore, the findings of this study can only be applied to the CAD patients without ≥50% diameter stenosis. The reason to exclude the patients with significant CAD was that the fall of blood pressure induced by ATP should have too much compromised coronary flow with the risk to produce an unwanted myocardial ischemia. Third, CVR assessed in the current study mean the resistance throughout the entire cardiac cycle, included the systolic period during which a compressive resistance is added to the resistance due to the coronary vasomotor tone, usually indicated as coronary “vascular” resistance (auto-regulative resistance).

## Conclusion

Serum EPA is significantly correlated with CFR in CAD patients without significant coronary artery stenosis.

## Competing interests

The authors declare that they have no competing interests.

## Authors’ contributions

SK designed the study protocol, carried out the MR studies, analyzed the data and drafted the manuscript. KF and JK analyzed the data and drafted the manuscript. NI, MK, YK, IK, TN, TN, YT, KU, KK and SU helped to draft the manuscript. All authors read and approved the final manuscript.
